# Age at Onset of Walking in Infancy Is Associated With Hip Shape in Early Old Age

**DOI:** 10.1002/jbmr.3627

**Published:** 2019-01-15

**Authors:** Alex Ireland, Fiona R Saunders, Stella G Muthuri, Anastasia V Pavlova, Rebecca J Hardy, Kathryn R Martin, Rebecca J Barr, Judith E Adams, Diana Kuh, Richard M Aspden, Jennifer S Gregory, Rachel Cooper

**Affiliations:** ^1^ School of Healthcare Science Manchester Metropolitan University Manchester UK; ^2^ Aberdeen Centre for Arthritis and Musculoskeletal Health School of Medicine Medical Sciences and Nutrition University of Aberdeen Aberdeen UK; ^3^ MRC Unit for Lifelong Health and Ageing at UCL London UK; ^4^ Medicines Monitoring Unit (MEMO) Division of Molecular & Clinical Medicine School of Medicine University of Dundee Ninewells Hospital & Medical School Dundee Scotland; ^5^ Manchester Academic Health Science Centre and Radiology Central Manchester University Hospitals NHS Foundation Trust and University of Manchester Manchester Royal Infirmary Manchester UK

**Keywords:** BIOMECHANICS, EXERCISE, BONE–MUSCLE INTERACTIONS

## Abstract

Bones’ shapes and structures adapt to the muscle and reaction forces they experience during everyday movements. Onset of independent walking, at approximately 12 months, represents the first postnatal exposure of the lower limbs to the large forces associated with bipedal movements; accordingly, earlier walking is associated with greater bone strength. However, associations between early life loading and joint shape have not been explored. We therefore examined associations between walking age and hip shape at age 60 to 64 years in 1423 individuals (740 women) from the MRC National Survey of Health and Development, a nationally representative British birth cohort. Walking age in months was obtained from maternal interview at age 2 years. Ten modes of variation in hip shape (HM1 to HM10), described by statistical shape models, were ascertained from DXA images. In sex‐adjusted analyses, earlier walking age was associated with higher HM1 and HM7 scores; these associations were maintained after further adjustment for height, body composition, and socioeconomic position. Earlier walking was also associated with lower HM2 scores in women only, and lower HM4 scores in men only. Taken together, this suggests that earlier walkers have proportionately larger (HM4) and flatter (HM1, HM4) femoral heads, wider (HM1, HM4, HM7) and flatter (HM1, HM7) femoral necks, a smaller neck‐shaft angle (HM1, HM4), anteversion (HM2, HM7), and early development of osteophytes (HM1). These results suggest that age at onset of walking in infancy is associated with variations in hip shape in older age. Early walkers have a larger femoral head and neck and smaller neck‐shaft angle; these features are associated with reduced hip fracture risk, but also represent an osteoarthritic‐like phenotype. Unlike results of previous studies of walking age and bone mass, associations in this study were not affected by adjustment for lean mass, suggesting that associations may relate directly to skeletal loading in early life when joint shape changes rapidly. © 2018 American Society for Bone and Mineral Research.

## Introduction

Hip geometry has been shown to be associated with a number of burdensome musculoskeletal health conditions including osteoarthritis (OA) and osteoporosis. Gross morphological features such as a wider femoral neck^1^ and femoral head,^2^ smaller neck‐shaft angle,^3^ and anteversion (external rotation of the femur)^4^ are associated with hip OA. Similarly, a narrower, longer femoral neck^5^ and greater neck‐shaft angle^6^ are associated with an increased risk of hip fracture. Hip shape is largely determined in childhood, although patterns vary between geometrical variables. Evidence suggests that neck‐shaft angle increases during gestation, and then decreases through childhood until around age 10,^7^ thereafter remaining stable throughout adulthood.^8^ Similarly, after peaking at birth, femoral neck anteversion declines dramatically in the first 3 to 4 years,^9^ thereafter declining more slowly until maturity. Increases in femoral neck length^10^, ^11^ and width^12^, and femoral head size^13^ occur until the late teens, although periosteal apposition continues to occur at a reduced rate throughout adulthood.^14^ Given the clinical relevance of these components of hip shape and their developmental timing, it is important to identify potentially modifiable factors in childhood that may influence hip shape.

One factor known to be closely related to hip development throughout childhood is mechanical loading of the skeleton during every day physical activity. The largest forces that routinely act upon the skeleton postnatally occur during bipedal movements such as walking and running, where whole‐body mass must be accelerated against gravity on a single limb. Onset of independent walking at around 1 year represents the first postnatal exposure to these locomotory forces. Accordingly, earlier‐walking infants have been shown to have advantages in bone geometrical measures in childhood.^15^ Population‐based epidemiological studies provide evidence to suggest that these advantages are evident through adolescence and into older age particularly in males, such that older men who started walking earlier have a larger hip area and femoral neck cross‐sectional area,^16^ as well as greater bone mass and BMD. With the exception of hip BMD, these associations remained robust to adjustment for body size (height, and lean and fat mass) and a number of possible confounders including birthweight and social class. Further evidence highlighting the importance of locomotory forces for skeletal development is found in studies of populations where these exposures are absent or delayed, such as in the case of individuals with cerebral palsy (CP), where walking age and broader motor development are delayed and physical activity is reduced. Children without CP have smaller femoral neck‐shaft angles and anteversion than affected children as measured by clinical and radiographic techniques, with group differences increasing as the level of motor impairment increases.^17^ However, to our knowledge no study has examined associations between age of walking onset (as a marker of early life skeletal loading) and detailed assessment of hip shape in older age.

Studies of hip geometry commonly report a single measure such as neck‐shaft angle or femoral neck length or width. However, hip geometry is a composite of these individual measures, which are often highly correlated. Statistical shape modeling (SSM) of the hip has been developed to objectively describe this coordinated variation in multiple components of shape through a series of shape modes. These models are very sensitive; differences in mode scores of only 0.2 to 0.3 SD have been shown to discriminate between hip OA^18^ and hip fracture^19^ cases and controls. Furthermore, SSM has clinical utility in predicting hip OA progression^20^ and in improving prospective prediction of hip fracture when combined with BMD measurements.^21^ With relevance to skeletal loading, recent work using SSM found associations between BMI at different stages of adulthood and hip shape in older men and women.^22^

To address important gaps in the existing literature, we examined associations between age at onset of independent walking and hip shape described by SSM in older men and women in a British birth cohort study: the Medical Research Council (MRC) National Survey of Health and Development (NSHD). It was hypothesized that earlier walking age would be associated with specific features of hip shape as described by SSM; namely wider femoral neck and head, and smaller neck‐shaft and anteversion angles as described in previous studies of mechanical loading in childhood.

## Participants and Methods

### Study population

The NSHD is a birth cohort study consisting of a socially stratified sample of 5,362 singleton births in 1 week in March 1946 in England, Scotland, and Wales. These participants have been prospectively followed regularly since birth. Between 2006 and 2010, eligible participants known to be alive and living in England, Scotland, and Wales were invited for an assessment at one of six clinical research facilities (CRFs). Of 2,856 individuals invited, 1690 attended a CRF and 539 received a home visit from a research nurse. Ethical approval for this data collection was obtained from the Central Manchester Research Ethics Committee (07/H1008/245) and the Scottish A Research Ethics Committee (08/MRE00/12).

### Hip DXA images

During the CRF assessment, images of the total body and hip were obtained using a QDR 4500 Discovery DXA scanner (Hologic, Inc., Bedford, MA, USA). Images were taken of the left hip, except in 63 cases where contraindication of a prosthesis in the left hip meant that the right hip was scanned. All hip scans were performed with the feet placed at 15 degrees of internal rotation. In five centers, scanners had rotating C‐arms allowing participants to lie supine for all scans; one center used a scanner with a fixed C‐arm. JEA's laboratory performed quantitative analyses of all scans and assessments for image quality. A manufacturer‐provided phantom was scanned daily prior to participant scanning; once a month, these results were sent to the coordinating center for scrutiny.

### Statistical shape modeling

Of the 1690 participants who attended a CRF, 1636 had a hip DXA scan. Three images were excluded because of extreme internal rotation of the femur, evidenced by femoral neck foreshortening, leaving 1633 images to build the hip SSM. SSM of hip images in this cohort has been described in detail previously.^22^ Briefly, a template of 68 points for the hip was placed around the area of interest in each image using Shape software (University of Aberdeen, Scotland). Procrustes transformation was used to translate, rotate, and scale the images to remove influences of size and alignment. Where osteophytes were evident on scans, they were manually marked using a series of five points (Supplementary Fig.  1). Principal component analysis was then performed to generate independent orthogonal modes of variation, describing in descending order percentage variation standardized to a mean of 0 and SD of 1. Ten modes were identified; each accounted for >2% hip shape variation. In total, these modes accounted for 80.6% of the total variance.^22^

### Age at onset of independent walking

The age in months at which their child first walked unaided was recalled by participants’ mothers during an assessment at age 2 years.

### Covariates

Potential confounders and mediators of the main associations between walking age and each hip mode were selected a priori based on existing literature.^16^, ^22^, ^23^ The potential confounders were birthweight, childhood socioeconomic position (SEP), adult SEP, and height, and the potential mediators were appendicular lean mass and appendicular fat mass. Birthweight was extracted from medical records within a few days of birth, and measurements to the nearest quarter‐pound (113 g) were converted to kilograms. As indicators of SEP, father's occupation at age 4 years (or at age 11 or 15 years if missing at age 4 years) and own occupation at age 53 years (or if not available, the most recent measure in adulthood) were both categorized into six groups (I = professional, II = managerial and technical, IIINM = skilled nonmanual, IIIM = skilled manual, IV = partly skilled, and V = unskilled) using the Registrar General's Social Classification.^24^ During the CRF visit, height in meters was measured, and appendicular lean and fat mass in kilograms were estimated from total‐body DXA scans.

### Statistical analysis

Data were analyzed using the R statistical environment (version 3.2.2; www.r-project.org), for 1423 participants (740 women) with hip shape mode data and all covariates. Associations between age at onset of independent walking and each hip shape mode were assessed using multiple linear regression models. There was no evidence of deviation from linearity when quadratic terms were included, so walking age (which was normally distributed) was modeled as a continuous linear variable. Walking age*sex interactions were examined given previous findings of sex‐specific associations of walking age with bone outcomes.^16^ Where sex interactions were identified (*P* < 0.1), subsequent models were sex‐stratified. Model 1 was adjusted for sex (unless sex‐stratified) and CRF location (as one CRF used a scanner with a fixed C‐arm requiring participants to be moved between scans). The impact of adjustment for each of the confounders and mediators identified above was then examined in turn before all covariates were entered into a final model (model 2) simultaneously.

In addition to describing associations between walking age and individual hip mode scores, we wanted to examine how overall hip shape described by these modes varied between earlier and later walkers. Therefore, we combined mean mode scores for early walkers (2 SD below the mean walking age = 9.0 months) and late walkers (+2 SD above the mean walking age = 18.5 months) for both women and men to generate mean hip shapes. The selection of these time points allowed us to visualize the majority of variance attributable to walking age as 95% of the population should be within 2 SD of the mean given a normal distribution. Given the linear associations between walking age and shape modes, smaller differences in walking age—1 SD—would result in a proportionately smaller difference in hip shape.

### Sensitivity analyses

To examine whether associations between walking age and hip shape were secondary to OA, DXA images were graded for hip OA and assigned Kellgren‐Lawrence grades as previously described.^25^, ^26^ Associations between walking age and OA were assessed in the whole cohort, then associations between walking age and hip shape modes were repeated in only the 707 individuals with no evidence of OA. Although there were no individuals with osteoporosis at the total hip site in this cohort (minimum hip BMD *Z‐*score was −2.4), we examined whether associations between walking age and hip shape were affected by adjustment for total hip BMD *Z*‐score. At age 13 years, the child's teacher graded their sporting ability as above average, average, or below average. This measure is used as an indicator of motor ability in later childhood, and is predictive of physical activity in adulthood.^27^ To assess whether associations were mediated by altered motor ability in later childhood, models were additionally adjusted for adolescent sporting ability in 1274 individuals for whom this information was available.

## Results

Characteristics of the participants included in this study are detailed in Table 1. Women were shorter and lighter than men, with lower appendicular lean and fat mass; there was also a tendency for women to have a lower birthweight than men. Women had lower scores than men for hip modes 1, 2, 4, 6, and 8, and higher scores for hip modes 3, 9, and 10 (Table 1).

**Table 1:** Characteristics of the MRC National Survey of Health and Development Stratified by Sex (Sample Restricted to Those With Complete Hip Shape Mode Data and Covariates)

	Women (*n* = 740)		Men (*n* = 683)		
Variable	Mean	SD	Mean	SD	Sex difference *P* value
Walking age (months)	13.6	2.3	13.7	2.3	0.40
Birthweight (kg)	3.39	0.62	3.45	0.57	0.06
	*n*	%	*n*	%	
Father's occupational class (age 4 years)					
I	55	7.4	60	8.8	0.74
II	172	23.2	151	22.1	
IIINM	142	19.2	134	19.6	
IIIM	204	27.6	197	28.8	
IV	131	17.7	104	15.2	
V	36	4.9	37	5.4	
Own occupational class (age 53 years)					
I	16	2.2	93	13.6	<0.01
II	313	42.3	326	47.7	
IIINM	265	35.8	76	11.1	
IIIM	44	5.9	141	20.6	
IV	77	10.4	39	5.7	
V	25	3.4	8	1.2	

Musculoskeletal assessments at 60 to 64 years					
	Mean	SD	Mean	SD	
Age at time of assessment (years)	63.2	1.1	63.2	1.2	0.13
Height (m)	1.62	0.06	1.75	0.06	<0.01
Weight (kg)	72.1	13.6	84.9	12.8	<0.01
Appendicular lean mass (kg)	16.2	2.5	24.6	3.4	<0.01
Appendicular fat mass (kg)	14.5	4.5	10	2.9	<0.01
Hip shape mode (HM) scores (and % of total variance)					
HM1 (23.0%)	−0.19	0.97	0.19	1	<0.01
HM2 (18.0%)	−0.19	0.96	0.19	1.02	<0.01
HM3 (11.9%)	0.21	0.92	−0.25	1	<0.01
HM4 (5.5%)	−0.2	0.9	0.22	1.06	<0.01
HM5 (5.4%)	−0.03	0.97	0.02	1.05	0.30
HM6 (5.3%)	−0.18	1	0.21	0.96	<0.01
HM7 (4.1%)	−0.03	1.01	0	0.96	0.54
HM8 (3.2%)	−0.15	0.97	0.13	1	<0.01
HM9 (2.3%)	0.13	0.98	−0.14	1	<0.01
HM10 (2.0%)	0.31	0.95	−0.36	0.93	<0.01

Earlier age at onset of independent walking was associated with greater HM1 and HM7 scores (Table 2) in model 1; these associations were not substantially affected by further adjustments in model 2. There was evidence of a sex interaction for HM2, with later walking age associated with greater scores in women only (*P* = 0.09 for sex interaction in both models). Conversely, later walking age was associated with greater HM4 scores in men only (*P* = 0.01 for sex interaction in both models). There was weak evidence of associations of later walking age with greater HM3 and HM6 scores (0.08 <* P* < 0.13 in both models). There was no evidence of associations between walking age and HM5 or HM8 to HM10 (*P* > 0.3 in all models).

**Table 2:** Associations Between Age at Onset of Independent Walking and Hip Shape Mode Outcomes in the MRC National Survey of Health and Development

Mode	Group	Model	Regression coefficient	95% CI		*P*	Sex interaction *P*
HM1	Combined	1	−0.036	−0.058	−0.014	<0.01	0.40
		2	−0.032	−0.055	−0.010	<0.01	0.55
HM2	Men	1	−0.014	−0.047	0.019	0.4	0.09
	Women		0.022	−0.008	0.052	0.16	
	Men	2	−0.001	−0.035	0.032	0.95	0.09
	Women		0.036	0.005	0.066	0.02	
HM3	Combined	1	0.016	−0.005	0.038	0.14	0.42
		2	0.019	−0.003	0.041	0.09	0.57
HM4	Men	1	0.042	0.008	0.076	0.01	0.01
	Women		−0.012	−0.040	0.016	0.39	
	Men	2	0.040	0.005	0.075	0.023	0.01
	Women		−0.006	−0.034	0.023	0.69	
HM5	Combined	1	−0.003	−0.025	0.020	0.82	0.53
		2	0.006	−0.017	0.029	0.63	0.56
HM6	Combined	1	0.02	−0.002	0.042	0.07	0.31
		2	0.02	−0.002	0.042	0.08	0.42
HM7	Combined	1	−0.032	−0.054	−0.009	<0.01	0.99
		2	−0.027	−0.050	−0.004	0.02	0.96
HM8	Combined	1	−0.005	−0.027	0.017	0.68	0.96
		2	−0.008	−0.031	0.015	0.50	0.98
HM9	Combined	1	−0.001	−0.023	0.021	0.95	0.33
		2	−0.005	−0.028	0.017	0.65	0.28
HM10	Combined	1	−0.01	−0.031	0.011	0.34	0.89
		2	−0.005	−0.027	0.016	0.63	0.7

Regression coefficients are the difference in mean HM scores per 1 month increase in walking age. Where sex interactions were evident (*P* for interaction < 0.1), sex‐specific associations are presented. Model 1 adjusted for sex (if men and women are combined) and CRF location. Model 2: model 1 + birthweight + father's occupational class + adult occupational class + height + appendicular fat mass + appendicular lean mass. Only results from basic and fully adjusted models are presented for brevity. When each set of covariates were adjusted for in turn, there was no evidence that any one specific set of factors was responsible for the attenuations observed between the models shown here.

It is important to note that shape modes are not measures of each separate component of hip shape, eg, neck‐shaft angle, femoral head, and neck size and shape. As components of hip shape tend to vary together, modes describe this covariation and hence each mode may reflect aspects of a number of different components. We use common terms to describe the features identified in each mode; a summary of these descriptions is provided in Supplementary Fig.  2. Taken together, in earlier walkers these modes describe a larger (HM4), flatter (less circular; HM1, HM4) femoral head, a wider (HM1, HM4, HM7), flatter (indicating a smoother transition from neck to head; HM1, HM7) femoral neck, smaller neck‐shaft angle (HM1, HM4), greater anteversion (HM2, HM7), and greater development of osteophytes (HM1).

**Figure 1:**
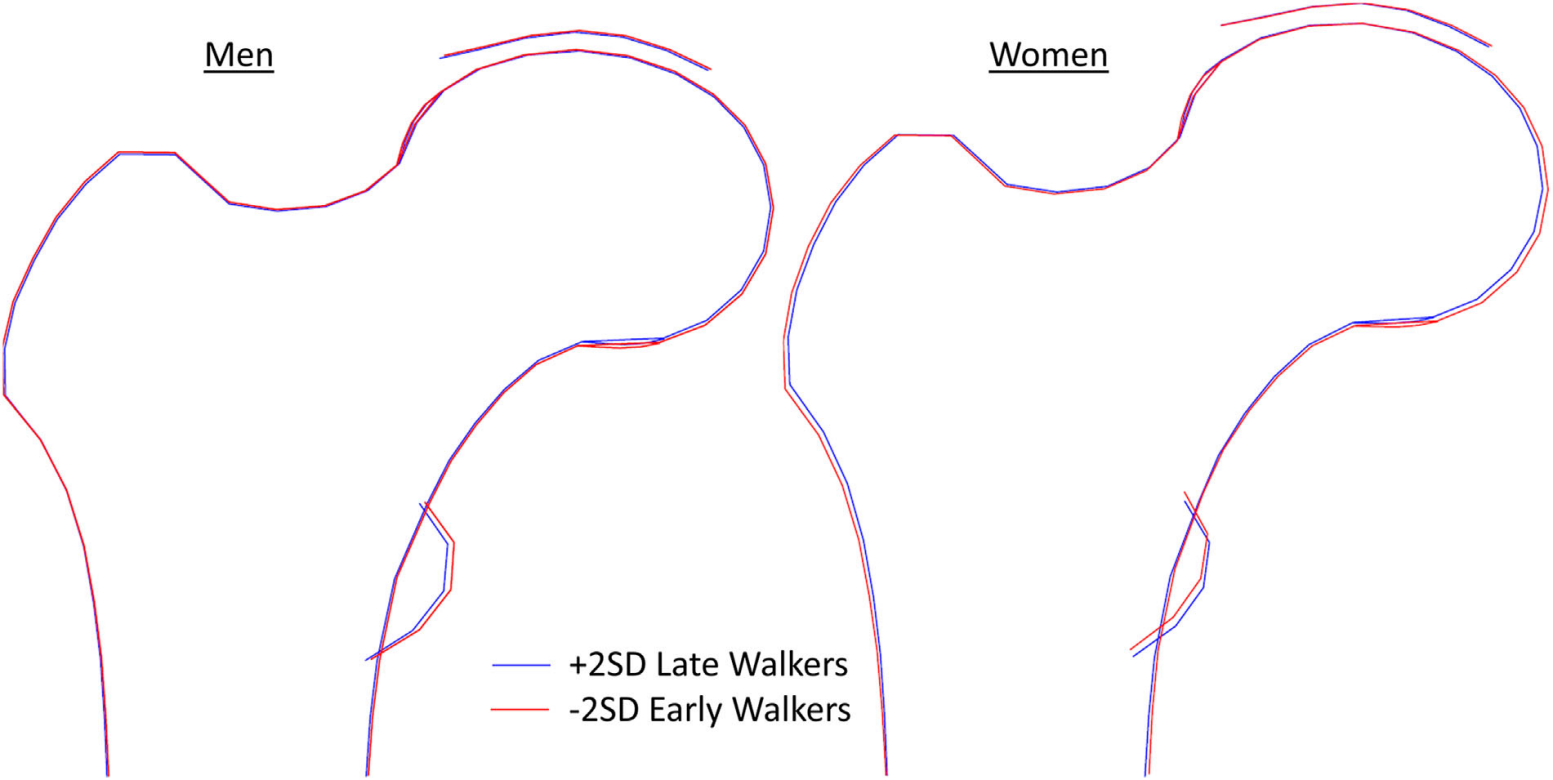
Mean hip shapes described by statistical shape models in early (−2 SD of the mean age) and late walking (+2 SD of the mean age) men and women. The mean age of walking in this cohort was 13.7 ± 2.3 months with no sex difference. Therefore, early and late walking as described above corresponded to walking at 9.0 months and walking at 18.5 months, respectively.

When combining mode scores for early and for late walkers we found that differences were very subtle (see Fig. 1). However, there was some suggestion that late walkers have a smaller femoral head, especially distally than early walkers in both sexes. In women, late walkers also had a smaller greater trochanter and a lesser trochanter displaced slightly distally from that of the late walkers, whereas late‐walking men had a smaller lesser trochanter with no apparent displacement. Early walkers showed evidence for variations in joint shape in regions of the superior and inferior borders of the femoral head where osteophytes commonly develop, which might indicate early osteophyte development.

### Sensitivity analysis

There was a low prevalence of hip OA in this cohort, as assessed by Kellgren‐Lawrence grading: 707 individuals (49.6%) were assigned grade 0 (no radiographic features of OA), 527 (37%) were grade 1 (possible joint space narrowing (JSN) and osteophytes), 168 (11.1%) were grade 2 (possible JSN with definite osteophytes), 20 (1.4%) were grade 3 (definite JSN, multiple osteophytes, sclerosis, and possible bony deformity), and only 1 individual was grade 4 (marked JSN, large osteophytes, severe sclerosis, and definite bony deformity). There was no association between walking age and Kellgren‐Lawrence grading. When analyses were restricted to the 707 individuals with no evidence of radiographic OA (Kellgren‐Lawrence grade 0), similar associations between walking age and hip shape mode scores were observed (results not shown). Similarly, there was no effect of further adjustment for total hip BMD *Z*‐score or sporting ability on associations between walking age and hip shape modes (results not shown).

## Discussion

The aim of this study was to examine associations between age at onset of independent walking and hip shape in older age, assessed by statistical shape models from regional DXA scans. Results showed a number of associations between walking age and hip modes. These associations were maintained after adjustment for a range of covariates including body size (height and body composition), and childhood and adult SEP. It is important to note that these differences in shape are independent of differences in hip bone size and mass previously observed in this cohort^16^ because of the Procrustes image scaling procedure performed prior to generation of shape modes.

In both sexes, early walking appears to be associated with a smaller neck‐shaft angle; this could be a consequence of a longer period of loading during a critical growing phase starting from the larger angle found in early infancy.^7^ In addition to a smaller neck‐shaft angle, earlier walking was also associated with higher scores for HM1 corresponding to a wider, shorter femoral neck, a flattening of the femoral head, and early development of osteophytes. Some of these features were also picked up by HM7, although this represented only 4% of the variance, and included some anteversion, as judged by the position of the lesser trochanter. These features in the hip shapes of earlier walkers are similar to those found to be associated with OA in several other studies.^20^, ^28^, ^29^, ^30^ Although this association is tantalizing, this group are still relatively young and show few signs of OA; it would be premature to say that earlier walking increases the risk of OA in early old age. However, these features have also been shown to be predictive of future OA development in a cohort of similar age with no evidence of radiographic OA at baseline.^20^ That similar associations between walking age and hip shape scores were found when analyses were restricted to individuals without radiographic OA suggests that these associations are not secondary to OA development. Conversely, however, later age at walking is associated with a longer, narrower femoral neck and a greater neck‐shaft angle. Together with the previous observations of smaller size and bone mass in men in this cohort,^16^ these are all features found to be risk factors for fracture of the femoral neck attributed to osteoporosis.^19^, ^21^, ^31^, ^32^ Although osteoporosis was prevalent at other sites in this cohort,^33^ there was no evidence of hip osteoporosis in individuals included in this study (minimum *Z*‐score −2.4) and few fractures; therefore, associations between walking age, hip shape, and fractures could not be explored. There was no effect of adjustment for hip BMD on associations between walking age and hip shape, although only weak associations between hip BMD and hip mode scores in this cohort have been reported.^22^ In addition, previous studies have shown that hip shape modes are predictive of hip fracture independent of BMD.^21^ The observed associations between walking age and hip shape do suggest that there may be links between motor development early in life and joint disorders in later life, but further studies are needed to explore this.

Sex‐specific variation was only identified for associations with HM2 and HM4. HM2, which was found to be associated with walking age in women but not in men, describes variations in shape almost as great as HM1, as shown by the variance explained; in this case, later walking was associated with a wider, shorter femoral neck, a large lesser trochanter, and no evidence for anteversion. For HM4, which was only associated with walking age in men, earlier walking was associated with a bigger, flatter femoral head, and wider femoral neck and smaller neck‐shaft angle, as shown by lower values for HM4.

Scores for individual shape modes are commonly standardized to a mean of 0 and SD of 1 to allow easier comprehension of how variance associated with predictors compares to variance in the broader population. In this study, differences in sex‐adjusted standardized hip mode scores between early walkers (walking age of 9 months) and late walkers (18.5 months) were 0.34 for HM1 and 0.30 for HM7, whereas female early walkers had 0.26 lower score for HM2 and male early walkers had 0.38 lower HM4 scores than late walkers. Although direct comparison of individual modes and scores cannot be made across cohorts, the magnitude of these differences is similar to that seen between healthy and clinical populations. For example, in a recent study using a similar SSM design, differences between cases and controls in hip shape modes associated with hip fracture were 0.21 for HM2 and 0.26 for HM4,^21^ whereas case‐control group differences in five hip shape modes associated with hip OA were 0.21 (HSM1), 0.31 (HSM3), 0.2 (HSM4), 0.24 (HSM8), and 0.19 (HSM10).^18^

Each mode in a statistical shape model identifies variations that occur in a coordinated pattern and that are linearly independent of the other modes. When recombined, some of these variations may counteract one another. This is both a strength and a weakness of the method. Its strength is that it can detect and isolate coordinated changes so that certain modes become indicative of pathology and can be used as imaging biomarkers.^18^, ^20^, ^21^, ^34^ The weakness, demonstrated here, is that the overall difference in shapes between early and late walkers, using all the modes in combination, is subtle, as indicated by the superimposed shapes in Fig 1. This is reflective of the relatively subtle interindividual variation in hip shape evident in Supplementary Fig. 2 (where shape difference representing 4 SD or 95% of total population variation are shown). Although differences are small, their effect is exerted over a lifetime; earlier walkers show more evidence of osteophyte development and a broadening distally of the femoral head. Both of these features are similar to those found in models of individuals with OA and maintain the possibility that earlier walking could be a risk factor for hip OA.

### Comparison with previous findings

To our knowledge, this is the first study to examine associations between walking age and joint shape in later life. Our findings extend those from previous studies of early walking and bone cross‐sectional shape in children, which found altered geometry in earlier‐walking toddlers^15^ with similar associations evident in adolescence.^35^ In previous analyses within the NSHD, we reported associations between early walking and greater femoral neck area assessed by standard DXA metrics.^16^ Our findings also agree with observations of faster childhood motor development being associated with smaller neck‐shaft angles.^17^ However, in previous studies early life loading is associated with reduced anteversion,^17^ whereas we observed associations between earlier walking and several modes indicating greater anteversion.

### Possible explanation of findings

Although physical activity can be beneficial for aspects of skeletal health such as bone mass accrual, it may be that high levels of specific types of activity at key periods of growth have undesirable effects on development of hip shape. For example, deformation of the femoral head is evident in elite sportspeople in adolescence.^36^, ^37^ This may relate to changes in growth plate shape and in skeletal loading patterns caused by different types of activity.^38^ These defects develop through adolescence and are related to initial hip shape.^39^ It may be that skeletal loading through walking during the first year of life (where skeletal growth is more rapid than at any other time point^40^) can have similar effects and this would be concordant with the reduced angulation, shortening, and flattening of the femoral neck and head observed in earlier walkers. Similarly, late walking may subject the growing hip to less active loading during a crucial phase in development and may leave the hip less than optimally formed and susceptible in later life to increased risk of fracture. However, little is known about hip and growth plate shape or skeletal loading patterns during early walking. Studies in this area could help explain the current findings and identify individuals and activities at high risk of adverse hip development.

In addition to direct effects of mechanical loading on bone development, previous studies suggest that altered childhood physical activity and lean mass may contribute to associations between walking age and bone outcomes.^16^, ^35^ This was supported by mediation of associations following adjustment for body composition, as a marker of active and passive skeletal loading. That similar adjustments did not explain the main associations in the current study may reflect the fact that hip shape is largely determined in childhood, with little change in adulthood unless affected by disease.^7^, ^8^, ^9^, ^10^, ^12^, ^13^, ^20^, ^41^ Whereas infant motor development is positively associated with adolescent physical activity, a previous study in this cohort found no associations between motor development and leisure time physical activity across adulthood.^42^ However, detailed information on early life physical activity and body composition was not available in this cohort; so we cannot investigate these potential mediating pathways, although adolescent sporting ability (as a marker of motor ability in later childhood) did not attenuate associations. Although periosteal apposition continues at a reduced rate through adulthood,^14^ the ability to increase skeletal size via exercise appears greatly diminished after maturity, whereas the ability to accrue bone mass is less affected.^43^ Studies of retired baseball players suggest that loading‐induced alterations in skeletal shape persist for several decades after exercise cessation.^44^ Therefore, it seems plausible that differences in hip shape directly related to mechanical loading through early walking may remain throughout life. Differences in early postnatal growth may have influenced associations, but between birth and age 2 years body size was not measured. However, previous associations between walking age and bone size and strength in early life are independent of differences in height and body mass.^15^

Sex‐specific associations with walking age were observed for two modes, with greater HM2 values in earlier‐walking women only and greater HM4 values in earlier‐walking men only. Greater HM2 values indicate external rotation and flattening of the femoral head, described in a number of other modes in both sexes. However, the mode also describes a longer femoral neck, which is an isolated finding not supported by other modes. Overall, men have larger HM4 values than women, which mainly relate to relative bone size (bigger femoral head and wider femoral neck), and these advantages were greater in earlier‐walking men. This sex‐specific association is similar to reports of greater hip area and femoral neck cross‐sectional area in earlier‐walking males only in a similar subcohort of the NSHD,^16^ and greater increases in bone size attributable to exercise in adolescent and adult males compared to females.^43^, ^45^

### Significance and implications

Whereas previous studies of walking age and bone have focused on bone mass and the implications for osteoporosis and fracture risk,^15^, ^16^, ^35^ this is the first study to consider detailed bone shape. Our findings not only reinforce the previous results regarding fracture risk, but may also be relevant to OA. The findings suggest an osteoarthritic‐like phenotype in earlier walkers, with features such as larger femoral neck^1^ and head,^2^ smaller neck‐shaft angle,^3^ and external rotation.^4^ Some of these features were previously described in a different cohort of older adults with hip OA using SSM.^20^ Conversely, the narrower, longer femoral neck^5^ and greater neck‐shaft angle^6^ evident in late walkers are associated with increased risk of hip fracture: These results are supported by a previous study using SSM in hip fracture cases and controls.^19^ Given the difficulty in launching prospective trials to examine a similar time period, examination of whether similar hip shape features are evident in earlier walkers in other cohorts would now be useful. Given the lack of effective pharmacological treatments for OA and the current downturn in prescriptions of effective osteoporosis drugs,^46^ the identification of novel modifiable factors influencing joint health is very timely.

If walking age is established as a predictor of hip shape through future studies, its utility is unclear. Onset of independent walking is highly variable and difficult to predict; in a study of 986 children, sex, ethnicity, SEP, and birth order only explained 2.5% of walking age variance.^47^ Motor development seems highly dependent on social influences as evidenced from differences in timing of motor milestones between geographical regions and cultures.^48^ A more detailed understanding of environmental factors predictive of precocious walking would help to identify individuals likely to walk early. Advantages in bone mass are not evident at birth in children who go on to walk earlier,^15^ but it is unknown whether bone shape differs between earlier and later walkers. Observational longitudinal studies of hip shape in children who walk at different ages would help us to understand at what stage the shape differences reported in this study first become evident, and whether they are in themselves predictive of walking development.

### Strengths and weakness

The strengths of this study include details of walking age collected six decades prior to skeletal health assessment, as well as a description of hip shape variation in a large population of older individuals of similar age, thereby removing the confounding effects of age. The associations examined in this study were adjusted for a number of potential confounders and mediators ascertained prospectively across life. The NSHD cohort has remained largely representative of the national population from which it was drawn despite losses to follow‐up.^49^ As an observational study, we cannot attribute causality. In addition, we have to consider the possibility that findings may be explained by residual confounding by factors not collected in this study, such as early childhood skeletal shape or childhood physical activity. Selection bias may also have influenced results; individuals who attended a clinic for DXA assessment were healthier and less likely to be obese than those who were visited at home.^50^

## Conclusions

In a relatively large nationally representative cohort, age at onset of independent walking was associated with differences in hip shape over 60 years later. In earlier walkers the identified shape features described an osteoarthritic phenotype characterized by features such as a flatter, larger femoral head, wider femoral neck, external rotation, smaller neck‐shaft angle, and possible osteophytosis. Hip features identified in later walkers described a shape associated with increased fracture risk, extending previous findings of low bone mass in this group. This supports a persisting role of early life mechanical loading in the development of joint shape features with clinical relevance in humans.

## Disclosures

All authors state that they have no conflicts of interest.

## Supporting information

Supporting Fig S1.

Supporting Fig S2.
