# Supplementary figures and images for: Age at Onset of Walking in Infancy Is Associated With Hip Shape in Early Old Age
Source: J Bone Miner Res. 2019 Jan 15;34(3):455–63. doi: 10.1002/jbmr.3627 (PMC6446733; doi:10.1002/jbmr.3627)

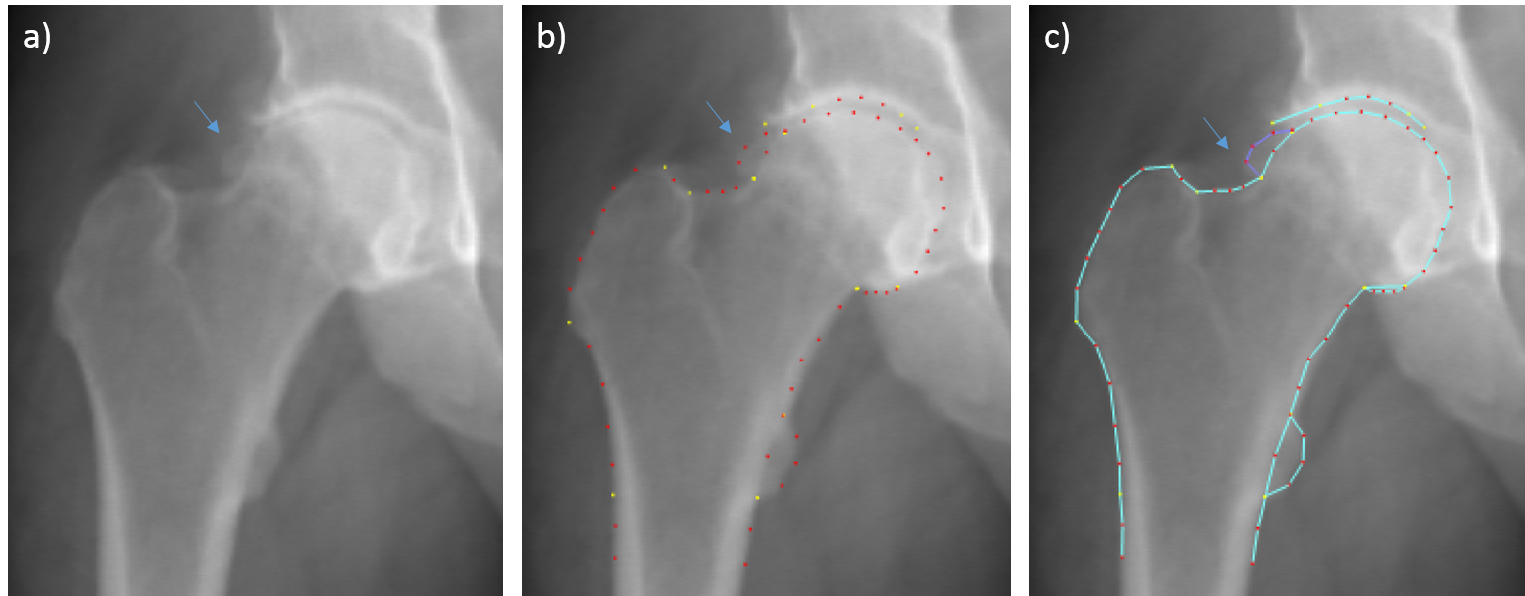

Supplement: Supplementary file 1 — Supporting Fig S1. [file JBMR-34-455-s001.tif]
