# Supplementary material for: Age at Onset of Walking in Infancy Is Associated With Hip Shape in Early Old Age
Source: J Bone Miner Res. 2019 Jan 15;34(3):455–63. doi: 10.1002/jbmr.3627 (PMC6446733; doi:10.1002/jbmr.3627)
Supplement: Supplementary file 2 — Supporting Fig S2. [file JBMR-34-455-s002.docx]

Supplementary Figure 2. Description of shape variation and associated features identified by hip modes HM1-HM10.

| Mode (% of variation) | Description | ±2 SD |
| --- | --- | --- |
| 1 (23.0%) | Negative scores   - More compact femoral head - Larger neck-shaft angle   Positive scores   - Femoral head migration - increased osteophytes - wider femoral neck - Smaller neck-shaft angle - Flattening of the femoral neck from negative to positive scores   *Hip Shape Modes with features similar to Positive scores above have been associated with increased risk of / severity of osteoarthritis in previous SSM studies^(19,21-23)^. Hip Shape Hip Shape Modes with features similar to Negative scores above have been associated with increased risk of fracture in previous SSM studies^(18,20,31,31)^.* |  |
| 2 (18.0%) | Negative scores   - Longer femoral neck - Increased external rotation, as shown by lesser trochanter inside the femoral shaft - Loss of femoral head curvature   Positive scores   - Wider greater trochanter - Larger lesser trochanter - Wider femoral head and neck - Superior and Inferior osteophytes   *Negative scores include some features (anteversion and reduced femoral head-neck curvature) previously associated with increased risk of osteoarthritis or total hip replacement in previous SSM studies^(19,29-31)^, albeit alongside a longer femoral neck* ***.*** *Due to the strict positioning protocols used with DXA, it is likely the apparent rotation is in fact variation in femoral anteversion.* |  |
| 3 (11.9%) | Negative scores   - Possible external rotation - Bigger femoral head - Loss of femoral head to neck curvature - Increased osteophytes superiorly and inferiorly - Wider femoral neck   Positive scores   - Smaller neck-shaft angle - Greater acetabular coverage |  |
| 4 (5.5 %) | Negative scores   - Bigger, flatter femoral head - Wider femoral neck - Smaller neck-shaft angle   Positive scores   - Possible external rotation - Increased inferior osteophytes - Small increase in acetabular coverage   *Hip Shape Modes with features similar to Negative scores above have been associated with increased risk of / severity of osteoarthritis in previous SSM studies^(19,29,30)^.* |  |
| 1. (5.4%) | Negative scores   - Possible external rotation (more of the lesser trochanter visible)   Positive scores   - Slight flattening of the inferior femoral head - Increased osteophytes |  |
| 1. (5.3%) | Negative scores   - Flattening of the femoral head - Change in curve between femoral head and neck - Some evidence of external rotation from positive to negative scores |  |
| 7 (4.1%) | Negative scores   - Longer femoral neck - More compact femoral head - Increase in osteophytes   Positive scores   - Wider, flatter femoral head - Shorter femoral neck - Slight external rotation   *Hip Shape Modes with features similar to Positive scores above have been associated with increased risk of / severity of osteoarthritis in previous SSM studies^(19,29,30)^.* |  |
| 8 (3.2%) | Negative scores   - Wider, flatter femoral head - Greater acetabular coverage - Larger superior osteophyte   Positive scores   - Slight medial migration of femoral head - Slightly larger lesser trochanter |  |
| 9 (2.3%) | Negative scores   - Wider femoral neck - Increasing osteophytes - More compact femoral head   Positive scores   - Slight proximo-medial migration of femoral head |  |
| 10 (2.0%) | Negative scores   - Flatter femoral neck curvature - Medial enlargement of femoral head - Narrower femoral shaft   Positive scores   - Wider greater trochanter - Greater acetabular coverage - Narrower neck |  |
